# Draft genome sequence of methicillin-resistant *Staphylococcus aureus* strain D1418m22 isolated from human wound pus

**DOI:** 10.1128/MRA.00409-23

**Published:** 2023-10-17

**Authors:** Daraksha Iram, Manish Singh Sansi, Anil Kumar Puniya, Sunita Meena, Shilpa Vij

**Affiliations:** 1 Antimicrobial Peptides, Biofunctional Probiotics & Peptidomics Laboratory, Dairy Microbiology Division, ICAR-National Dairy Research Institute, Karnal, Haryana, India; 2 Biofunctional Peptidomics & Metabolic Syndrome Laboratory, Animal Biochemistry Division, ICAR-National Dairy Research Institute, Karnal, Haryana, India; 3 Anaerobic Microbial Fermentation Laboratory, Dairy Microbiology Division, ICAR-National Dairy Research Institute, Karnal, Haryana, India; University of Maryland School of Medicine, Baltimore, Maryland, USA

**Keywords:** MRSA, WGS, GO

## Abstract

This study reveals the complete genome sequence of methicillin-resistant *Staphylococcus aureus* (MRSA) strain d1418m22, sourced from a Karnal, Haryana, human skin wound. Classified as community-associated MRSA, it features a 2.78-MB genome harboring *Staphylococcus*-specific genetic elements, encompassing 2,625 protein-coding genes and 18 antimicrobial resistance genes.

## ANNOUNCEMENT

The coagulase-positive pathogenic strains of *Staphylococcus aureus* are recognized worldwide as zoonotic and multidrug-resistant opportunistic pathogens that can cause both nosocomial and community-acquired illnesses (1, 2). Through horizontal gene transfer of the staphylococcal chromosome mec (SCCmec), methicillin-resistant *S. aureus* (MRSA) acquires drug resistance to multiple beta-lactam antibiotics, including methicillin, oxacillin, penicillin, amoxicillin, and cephalosporins (3).

There are multiple SSCmec genotypes, with SSCmec types I–III found in hospital-acquired *S. aureus* isolates and types IV and V in community-acquired isolates. Types I–III typically encode resistance to multiple antibiotics, including methicillin. Types IV–V only encode resistance to methicillin (4, 5). Then, we report the genome sequence of a hospital-acquired MRSA isolate.

The MRSA strain (d1418m22) was isolated from a human skin wound in Karnal, Haryana, India. The culture was grown for 16 to 20 h at 37°C in Muller Hinton broth media (Himedia, Mumbai, India). Genomic DNA was extracted with the AlexGen bacterial gDNA extraction kit (Alexius Biosciences, Germany) according to the manufacturer’s instructions. Genomic DNA libraries were prepared using a QIAseq FX DNA Library UDI-B Kit (QIAGEN, Hilden, Germany) following the manufacturer’s protocol with the following modifications: 5  ng of DNA was used as the input, and the PCR elongation time was increased from 30 s to 1  min. Libraries were sequenced on the Illumina Novaseq 6000 platform using a 300-bp paired-end read protocol. A high-fidelity amplification step was completed using HiFi PCR Master Mix. The high-sensitivity D1000 ScreenTape was used to analyze the amplified libraries on the TapeStation 4150 (Agilent Technologies). *De novo* assembly was performed with KmerGenie (6), Velvet (7), SPAdes (8), and GapFiller (9). The final draft genomes were estimated to be 100.0% complete by using BUSCO (version 5.4.6) software (10). The default settings were utilized for all software. The present study identified a total of 20,229,752 high-quality reads, with a guanine-cytosine content of 32.72%. *De novo* assembly yielded 61 scaffolds, with a N50 of 130,908 bp, a maximal scaffold size of 290,662 bp, and a genome size of 2.78 Mb, which is comparable to the genome length of previous sequenced MRSA strains (11, 12). Additionally, the Prodigal software predicted 2,625 protein-coding genes across all scaffolds. Then, proteins were annotated against the NCBI nonredundant database by using BlastP, with an *E*-value of 1e^−6^, and genes (1,961) were confirmed to be homologous to *S. aureus*. There were 1,286 Gene Ontology (GO) terms assigned to biological processes, 423 GO terms associated with cellular components, and 1,466 GO terms linked to molecular functions. Additionally, a homology search using the BLASTP program and an *E*-value threshold of 1e^−5^ were performed to compare the proteins in the assembled *S. aureus* genome to antibiotic resistance genes collected from the comprehensive antibiotic resistance database. Overall, a total of 18 antimicrobial resistance genes were identified.

## Data Availability

The assembled de novo whole-genome sequences were deposited at GenBank (accession number JAPDDY000000000), with general details available under BioProject identification number PRJNA894174, BioSample number SAMN31439881, and SRA project number SRR24389989. The project’s approval number by the Institutional Biosafety Committee (IBSC) was 11-2021/IRC D 61.
